# Deep-Learning-Based Physical Layer Authentication for Industrial Wireless Sensor Networks

**DOI:** 10.3390/s19112440

**Published:** 2019-05-28

**Authors:** Run-Fa Liao, Hong Wen, Jinsong Wu, Fei Pan, Aidong Xu, Yixin Jiang, Feiyi Xie, Minggui Cao

**Affiliations:** 1 National Key Laboratory of Science and Technology on Communications, University of Electronic Science and Technology of China, Chengdu 611731, China; runfa.liao@std.uestc.edu.cn (R.-F.L.); panfeivivi@hotmail.com (F.P.); helloyuiki@foxmail.com (F.X.); cmjcmjcmj@163.com (M.C.); 2 School of Aeronautics and Astronautics, University of Electronic Science and Technology of China, Chengdu 611731, China; 3 Department of Electrical Engineering, Universidad de Chile, Santiago 8370451, Chile; wujs@ieee.org; 4 EPRI, China Southern Power Grid Co., Ltd., Guangzhou 510080, China; xuad@csg.cn (A.X.); jiangyx@csg.cn (Y.J.)

**Keywords:** PHY-layer, light-weight authentication, neural network, WSN, industrial

## Abstract

In this paper, a deep learning (DL)-based physical (PHY) layer authentication framework is proposed to enhance the security of industrial wireless sensor networks (IWSNs). Three algorithms, the deep neural network (DNN)-based sensor nodes’ authentication method, the convolutional neural network (CNN)-based sensor nodes’ authentication method, and the convolution preprocessing neural network (CPNN)-based sensor nodes’ authentication method, have been adopted to implement the PHY-layer authentication in IWSNs. Among them, the improved CPNN-based algorithm requires few computing resources and has extremely low latency, which enable a lightweight multi-node PHY-layer authentication. The adaptive moment estimation (Adam) accelerated gradient algorithm and minibatch skill are used to accelerate the training of the neural networks. Simulations are performed to evaluate the performance of each algorithm and a brief analysis of the application scenarios for each algorithm is discussed. Moreover, the experiments have been performed with universal software radio peripherals (USRPs) to evaluate the authentication performance of the proposed algorithms. Due to the trainings being performed on the edge sides, the proposed method can implement a lightweight authentication for the sensor nodes under the edge computing (EC) system in IWSNs.

## 1. Introduction

With the development of Industry 4.0, wireless sensor networks (WSNs) have great application prospects for industrial scenarios due to their advantages over traditional wired networks [1,2,3,4]. However, fully-automated mechanized operations and the wireless communication environments make the industrial wireless sensor networks (IWSNs) have stronger requirements for high security and low latency [5]. M.Luvisotto et al. [6] mentioned that the response delay in IWSNs should be in milliseconds. Moreover, under the edge computing (EC) system in IWSNs, some sensor nodes are in some completely security-free environments because there are no redundant computing resources and transmission resources. Therefore, lightweight authentication is urgently needed to enhance the security of IWSNs while ensuring low latency. The encrypted methods [7,8] are too heavy to support the nodes due to complex computing. I. Bhardwaj et al. [9] did some lightweight processing on the password, but their method still cannot meet some specific requirements. Some other researchers proposed a fast cross-authentication scheme that combines non-cryptographic and cryptographic algorithms to solve the security and latency issues [10]. In addition, the heterogeneous nature of the IWSNs makes traditional encryption-based authentication methods more complex to implement or manage. However, physical (PHY) layer methods provide some new approaches to protect the lightweightIWSNs. The high authentication rate and low cost are especially valued for such applications. By introducing deep learning (DL) into the PHY-layer authentication method, under the EC system, the training is performed under the edge devices, and the sensor nodes almost do not bear any extra costs.

D. Christin et al. [1] surveyed related WSN technologies dedicated to industrial automation from the aspects of security and quality of service (QoS). The work in [4] presented a QoS framework for IWSNs guaranteeing the delay bound and the target reliability. N. Neshenko et al. [11] surveyed the challenges and research problems in the Internet of Things (IoT) including intrusion detection systems, threat modeling, and emerging technologies. However, the papers mentioned above only address the security and reliability issues from the perspective of the system architecture or simply give a direction for future research. L. Xiao et al. [12] proposed a method to enhance the security of underwater sensor networks exploiting the power delay profile of the underwater acoustic channel to discriminate the sensors. The article [13] presented a two-factor user authentication protocol using the hash function that protects against other attacks in wireless sensor networks, with the exception of denial of service (DoS) and node attacks. However, the traditional security methods have relatively large requirements on computing resources and communication resources, which cannot meet the requirement of low latency.

PHY-layer authentication can achieve lightweight authentication and effectively address the tradeoff between the security and low latency requirement of the wireless sensor networks in industrial scenarios. The PHY-layer authentication methods can distinguish the legitimate sensor nodes and illegal ones by physical layer channel information, such as channel state information (CSI) [14,15,16,17], received signal strength indicator (RSSI) [18,19,20], received signal strength (RSS) [21], and the radio frequency (RF) fingerprint [22,23]. However, the PHY-layer authentication methods mentioned above based on the hypothesis test are mostly compared with a threshold to distinguish users, which makes it difficult to discriminate multi-nodes at the same time. Authenticating multi-nodes simultaneously is a multi-classification problem, which needs to be solved urgently.

Deep learning has a large number of applications, such as computer vision, image classification, pattern recognition [24,25,26], and so on. There are considerable research works using deep learning in wireless communications, such as in channel estimation and channel prediction. P. Illy et al. used machine learning to enhance the security of edge computing by implementing intrusion detection [27]. The paper [28] used the deep neural network to estimate the CSIs in orthogonal frequency division multiplexing (OFDM) systems. The work in [29] proposed a Raleigh fading channel prediction scheme with a deep learning method. N. Wang et al. [30] proposed a physical-layer authentication scheme based on extreme learning machine to detect spoofing attack. The DL-based PHY-layer authentication methods proposed in this paper can achieve multi-user authentication in a short time.

Unlike the traditional test-threshold-based PHY-layer authentication, the DL-based PHY-layer authentication methods can distinguish multiple sensor nodes simultaneously and maintain excellent performance. In the EC system, multi-sensor nodes need to be authenticated simultaneously, which is suitable for using the DL-based methods. The DL-based authentication methods are usually divided into the offline training phase and online authentication phase. The PHY-layer authentication framework we proposed in this paper also includes an online retraining process. In summary, the DL-based sensor nodes’ authentication algorithms proposed in this paper, utilizing the spatial diversity of wireless channels, can discriminate the sensor nodes without the test thresholds and have more practical application values. The main contributions of our work can be summarized as follows:

- We propose a DL-based PHY-layer authentication framework to enhance the security of industrial sensor networks. We also briefly explore the applications of the framework for practical industrial scenarios.
- Three different algorithms are adopted to implement the PHY-layer authentication in IWSNs, including the deep neural network (DNN)-based sensor nodes’ authentication method, the convolutional neural network (CNN)-based sensor nodes’ authentication method, and the convolution preprocessing neural network (CPNN)-based sensor nodes’ authentication method.
- Simulation results show that the proposed algorithms can achieve better performance. In addition, the experiments in the engineering center with USRPs validate their utility in practical industrial environments.

The rest of this paper is organized as follows. We present the preliminaries and system model in Section 2 and Section 3, respectively. The DL-based PHY-layer authentication method in industrial wireless sensor networks is proposed in Section 4. We provide numerical experiments in Section 5. The experiment in a practical environment and conclusions are presented in Section 6 and Section 7, respectively.

The symbols used in this article are briefly described as follows. Uppercase bold letters are used for the matrix (e.g., H, W) and lowercase bold letters for vectors (e.g., x, y). The elements are represented by the letters with subscripts and not bold (e.g., $x_{i}$, $\omega_{1i}$).

## 2. Preliminaries

### 2.1. Channel State Information

Due to the inherent characteristics of the wireless channels, the transmitted signals may experience a series of attenuations, such as, multipath effects, fading, shadowing, and delay distortion. The channel state information (CSI) provides us the channel variations experienced during propagations. In wireless communications, CSI represents the channel properties of a communication link. The CSI needs to be estimated by the receiver to detect the transmitted signals.

In the wireless fading channel, the system is modeled as:

(1) y=Hx+n,

where y and x represents the receive and transmit signal, respectively. H denotes the channel matrix, which is the CSI we mentioned above. n denotes the additive white Gaussian noise vector, which follows a complex standard normal distribution. $n∼CN\left(0,\sigma_{0}\right)$, where the mean value is zero and the noise covariance matrix $\sigma_{0}$ is known. H represents the channel’s frequency response, which can be estimated by y and x in the receiving end.

### 2.2. Deep Neural Network

**Figure 1:**
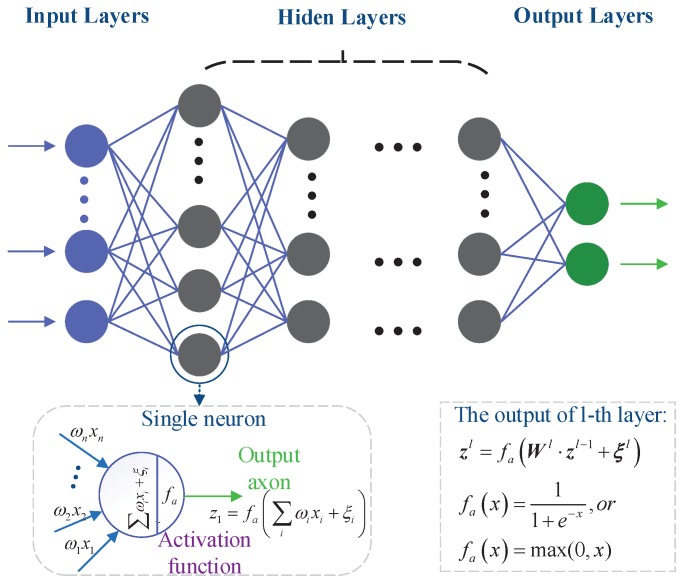
The deep neural network.

Generally speaking, DNN is a deeper version of the artificial neural network (ANN) through increasing the number of hidden layers in order to enhance the ability in representation or classification. As shown in Figure 1, it is a typical deep neural network with an input layer, multiple hidden layers, and an output layer. Each layer has a large number of neurons. The input of each neuron is the output of the upper neuron multiplied by the corresponding coefficient, and the output of each neuron is the input activated by activation functions. For example, the output of the first neuron in the first hidden layer is:

(2) $$z_{1}^{1}=f_{a}\left(\sum_{i}\omega_{1i}x_{i}+\xi_{1}\right),$$

where $\omega_{1i}$ denotes the weight coefficient of links $z_{1}^{1}$ and $x_{i}$. $\xi_{1}$ denotes the threshold coefficient of $z_{1}^{1}$. $f_{a}\left(\cdot \right)$ represents the activation function. Common activation functions are the sigmoid function, the rectified linear unit (ReLU) function, and the soft-max function, defined as $f_{sigmoid}\left(x\right)=\frac{1}{1-e^{-x}}$, $f_{ReLU}\left(x\right)=\max\left(0,x\right)$, $f_{softmax}\left(x\right)=\frac{e^{x}}{{∥e^{x}∥}_{1}}$, respectively, where x is a vector and ${∥\cdot ∥}_{1}$ denotes the $\ell_{1}$-norm. Usually, the hidden layer and output layer use the ReLU function and the soft-max function, respectively. The output of $l^{\mathrm{th}}$ layer is given by:

(3) $$z^{l}=f_{a}^{l}\left(W^{l}\cdot z^{l}+\xi^{l}\right).$$

We use the $\Psi^{l}\left(\cdot \right)$ to represent the operation of each layer of neurons. Then, we have the output of the deep neural network,

(4) $$\hat{y}=\Psi \left(W,\Xi \right)=\Psi^{L}\left(\Psi^{L-1}\left(\ldots \Psi^{1}\left(x\right)\right)\right).$$

The application of the neural network is executed in two steps, a training phase and an identification phase. When in the training phase, the input data (i.e., CSI) of the input layer and the corresponding label y are known. Then, we train the parameters W and ξ by minimizing the cost function L by the gradient descent method, which is formulated as:

(5) $$\hat{W},\hat{\xi }=\arg\min_{W,\xi }\left(L\right),$$

where L represents the value of the loss function. The loss function usually uses a mean squared error function or a cross entropy function, which is given by:

(6) $$L_{mean-square}={∥y-\hat{y}∥}_{2}^{2},$$

or:

(7) $$L_{cross-entropy}=y^{T}\cdot \log\left(\hat{y}\right),$$

where ${\left(\cdot \right)}^{T}$ denotes the transpose of the matrix or vector.

In the identification phase, the label of the input data (i.e., CSI) is unknown. By inputting CSI to the neural network, its corresponding output $\hat{y}$ will be used to identify and classify the input CSI.

### 2.3. Convolutional Neural Network

The convolutional neural network (CNN) is part of the feedforward neural network with convolutional computation and a deep structure [11]. CNN includes convolutional layers, pooling layers, and fully-connected layers compared with ordinary neural network. The convolutional layer computes multiple convolutions in parallel to produce a set of linear activation responses. Further, the convolution operation can effectively extract features form the original signal (e.g., CSI). The output of the convolutional layer is given by:

(8) $$Z^{l}=f_{a}^{l}\left(Z^{l-1}⊗W^{l}+\xi^{l}\right),$$

where $Z^{l}$ denotes the output of the $l^{\mathrm{th}}$ layer and $W^{l}$ and $\xi^{l}$ denote the convolution kernel and threshold in the $l^{\mathrm{th}}$ layer, respectively. ⊗ represents the convolution operation. $f_{a}^{l}\left(\cdot \right)$ denotes the activation function in CNN, often using the ReLU function.

Following the convolutional layer is the pooling layer, which effectively reduces the data dimension without losing valid information. The pooling function replaces the output of the network at that location using the overall statistical characteristics of the adjacent outputs at a location; for example, the maximum value in the adjacent rectangular region. Other commonly-used pooling functions include the average value in an adjacent rectangular region, the $\ell_{2}$ norm, and the weighted average in adjacent regions. The main goal is to reduce the dimension or the resolution of feature maps. The pooling operation, which is a subsampling, can facilitate the extraction of high-level features.

**Figure 2:**
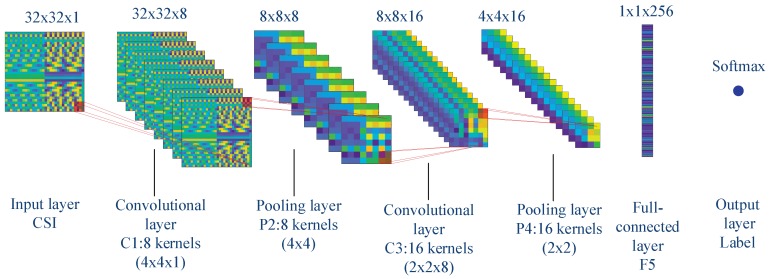
The convolutional neural network. CSI, channel state information.

The fully-connected layer of CNN is more like a hidden layer in DNN. There can be one fully-connected layer or multiple in CNN. We convert CSI into a matrix and use different colors to represent different values. As shown in Figure 2, it is a typical convolutional neural network with two convolutional layers, two pooled layers, and one fully-connected layer. We can see that the CSI converts to a matrix of 32 by 32 in size. The size of the convolution kernel in the first convolutional layer is four by four. After the convolution and activation, the average pooling operation is performed with a kernel of four by four in size. Then, there is another convolution, activation, and pooling operation. The final two layers are the fully-connected layer and the output layer activated with soft-max. The output of CNN can be formulated as:

(9) $$\hat{y}=\Upsilon \left(w,\xi \right)=\Upsilon^{L}\left(\Upsilon^{L-1}\left(\ldots \Upsilon^{1}\left(X\right)\right)\right).$$

Like DNN, CNN is also executed in two steps, a training phase and an identification phase. During the training phase, the input data (i.e., CSI) and corresponding labels y will be used to train the parameters w and ξ in CNN, which is formulated as:

(10) $$\hat{w},\hat{\xi }=\arg\min_{w,\xi }\left(L\right),$$

where L denotes the value of the loss function in CNN. In the identification phase, the well-trained CNN will be used to perform the PHY-layer authentication.

## 3. System Model

**Figure 3:**
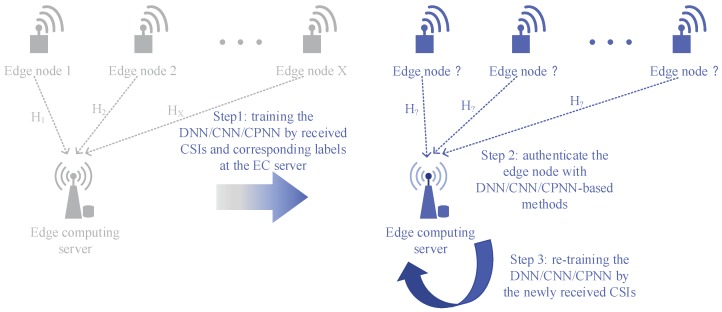
The system model of DL-based PHY-layer authentication in IWSNs.

**Figure 4:**
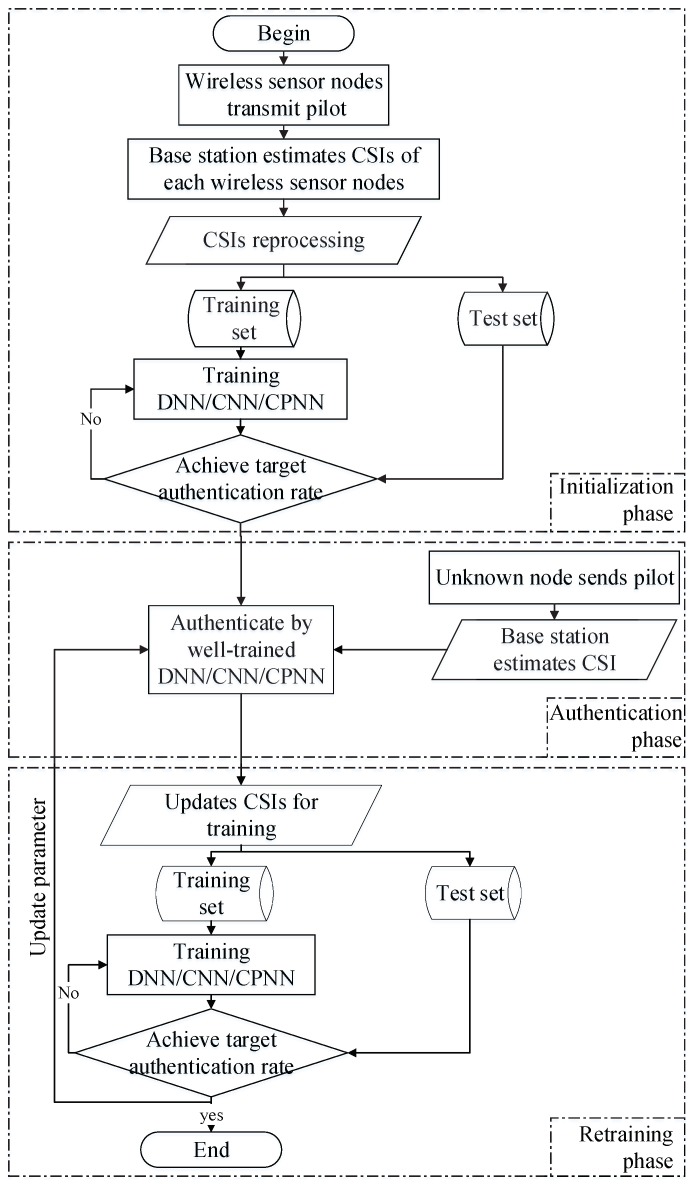
DL-based PHY-layer authentication flow chart.

We propose a DL-based PHY-layer authentication for an industrial wireless sensor network that can resist the spoofing attack. The methods we propose can enhance the security of the industrial wireless network without sacrificing communication resources. As shown in Figure 3, we placed many sensor nodes in the different locations of the industrial scene. The wireless sensor nodes send the pilot to the base station (BS) with time division duplexing (TDD) mode. First of all, each node needs to be identified by the upper layer authentication to facilitate labeling the corresponding CSI. In the initialization phase, we trained our neural networks through the training data (i.e., CSIs) and corresponding labels. Then, we authenticated the legitimate and illegal sensor nodes with newly-estimated CSI in the authentication phase. In the retraining phase, we updated the CSIs’ training set with the new channel information of certified sensor nodes and retrained the neural network for the next authentication. The authentication processing of the industrial wireless sensor network is shown in Figure 4.

The DL-based PHY-layer authentication we propose can dynamically adjust system parameters over time. It can further improve the accuracy of authentication and has higher practicality.

## 4. Deep Learning-Based Sensor Nodes’ Authentication Algorithms

In our previous work, we briefly introduced the physical layer channel authentication based on CNN [31]. This paper will further improve the CNN algorithm and propose a rapid-DNN-based PHY-layer authentication algorithm to meet the low latency requirements of industrial wireless sensor networks.

### 4.1. DNN-Based Sensor Nodes’ Authentication

The DNN-based PHY-layer authentication in industrial wireless sensor networks uses the DNN to implement sensor nodes’ authentication. In the initialization phase, the base station collects channel state information of each sensor node and performs the corresponding labeling according to the upper layer protocol authentication (e.g., EAP, AKA). The DNN was trained by the collected information to obtain the initial neural network parameters. In the authentication phase, the CSI of the unknown sensor node will be authenticated by the well-trained DNN in the initialization phase. After the new CSI has been authenticated, the dataset will be trained again for the next authentication.

**Algorithm 1** DNN-based sensor nodes’ authentication.**Input:** The $i^{\mathrm{th}}$ CSI to authenticate $x^{\left(i\right)}$**Output:** The label of unknown CSI ${\hat{y}}^{\left(i\right)}$, the new weight matrix $W^{†}$, and threshold vector $\xi^{†}$ of DNN1: Initialize all connection weights $W_{0}^{†}$, and thresholds $\xi_{0}^{†}$ in the network will be obtained through the training of DNN, using the pre-acquired dataset $D^{†}={\left\{\left(x_{k},y_{k}\right)\right\}}_{k=1}^{m}$;2: Compute ${\hat{y}}^{\left(i\right)}$ by well-trained DNN;3: Update the training set $D^{†}={\left\{\left(x_{k},y_{k}\right)\right\}}_{k=1}^{m}$ by $\left(x^{\left(i\right)},{\hat{y}}^{\left(i\right)}\right)$;4: Retrain the DNN by the new dataset and get new weight matrix $W^{†}$ and threshold vector $\xi^{†}$;5: Return ${\hat{y}}^{\left(i\right)}$, $W^{†}$, $\xi^{†}$.

### 4.2. CNN-Based Sensor Nodes’ Authentication

The CNN-based sensor nodes’ authentication method is more like the DNN-based sensor nodes’ authentication. They have the same steps except that the authenticated neural network changes from DNN to CNN. In the initialization phase, the CNN will be trained by the pre-acquired dataset of different sensor nodes. Then, the $i^{\mathrm{th}}$ CSI will be authenticated by the well-trained CNN. At last, the CNN will be retrained after the dataset is updated.

**Algorithm 2** CNN-based sensor nodes’ authentication.**Input:** The $i^{\mathrm{th}}$ CSI to authenticate $x^{\left(i\right)}$**Output:** The label of unknown CSI ${\hat{y}}^{\left(i\right)}$, the new weight matrix $W^{◊}$, and threshold vector $\xi^{◊}$ of CNN1: Initialize all connection weights $W_{0}^{◊}$, and thresholds $\xi_{0}^{◊}$ in the network will be obtained through the training of CNN, using the pre-acquired dataset $D^{◊}={\left\{\left(x_{k},y_{k}\right)\right\}}_{k=1}^{m}$;2: Compute ${\hat{y}}^{\left(i\right)}$ by the well-trained CNN;3: Update the training set $D^{◊}={\left\{\left(x_{k},y_{k}\right)\right\}}_{k=1}^{m}$ by $\left(x^{\left(i\right)},{\hat{y}}^{\left(i\right)}\right)$;4: Retrain the CNN by the new dataset and get new weight matrix $W^{◊}$ and threshold vector $\xi^{◊}$;5: Return ${\hat{y}}^{\left(i\right)}$, $W^{◊}$, $\xi^{◊}$.

### 4.3. Convolution Pre-Processing Neural Network-based Sensor Nodes’ Authentication

The convolution pre-processing neural network-based sensor nodes’ authentication method we propose in this paper has shorter training time and higher authentication accuracy. The core idea is to perform offline convolution preprocessing on the CSIs before training the neural network. The convolution preprocessing can effectively reduce the data dimension and extract the feature information of the CSIs, while the convolution kernels are trained by pre-obtained CSIs and corresponding labels. After convolution, activation, and pooling by the convolution kernels, the CSIs $x_{k}$ become ${\bar{x}}_{k}$. The latter’s dimensions are much smaller than the former’s. For the CPNN-based sensor node authentication method, the convolution kernels $V^{⊥}$ need to be calculated by the pre-obtained CSIs. Then, the neural network is trained by the new dataset $D^{⊥}={\left\{\left({\bar{x}}_{k},y_{k}\right)\right\}}_{k=1}^{m}$ in the initialization phase to get the weight matrix $W_{0}^{⊥}$ and threshold vector $\xi_{0}^{⊥}$.

**Algorithm 3** CPNN-based sensor nodes’ authentication.**Input:** The $i^{\mathrm{th}}$ CSI to authenticate $x^{\left(i\right)}$**Output:** The label of unknown CSI ${\hat{y}}^{\left(i\right)}$, the new weight matrix $W^{⊥}$, and threshold vector $\xi^{⊥}$ of CPNN1: Initialize: training the CNN by the pre-acquired CSIs to obtain the convolution kernels $V^{⊥}$; the dataset $D^{⊥}={\left\{\left({\bar{x}}_{k},y_{k}\right)\right\}}_{k=1}^{m}$ obtained by convolution; the weights $W_{0}^{⊥}$ and thresholds $\xi_{0}^{⊥}$ in the neural network will be obtained through the training of CPNN, using dataset $D^{⊥}={\left\{\left({\bar{x}}_{k},y_{k}\right)\right\}}_{k=1}^{m}$;2: Convolution pre-processing of the CSI $x^{\left(i\right)}$ into ${\bar{x}}^{\left(i\right)}$;3: Compute ${\hat{y}}^{\left(i\right)}$ by the well-trained CPNN;4: Update the training set $D^{⊥}={\left\{\left({\bar{x}}_{k},y_{k}\right)\right\}}_{k=1}^{m}$ by $\left(x^{\left(i\right)},{\hat{y}}^{\left(i\right)}\right)$;5: Retrain the CPNN by the new dataset, and get new weight matrix $W^{⊥}$ and threshold vector $\xi^{⊥}$;6: Return ${\hat{y}}^{\left(i\right)}$, $W^{⊥}$, $\xi^{⊥}$.

### 4.4. Complexity Analysis

We compare the computational complexity of each sensor nodes’ authentication methods in this section. The initialization phase was performed offline, and we will not discuss its computational resources and latency issues. In the authentication phase, the DNN-based sensor nodes’ authentication method needs to perform:

(11) $$b^{l}=W^{l}\cdot z^{l-1}+\xi^{l}.$$

As shown in Table 1, the computational complexity of the mathematical operation of DNN-based method is almost $O\left(\max\left(n^{1}\times n^{2},n^{2}\times n^{3},\ldots ,n^{L-1}\times n^{L}\right)\right)$, where $n^{l}$ denotes the number of neurons in the $l^{\mathrm{th}}$ layer in DNN. In our numerical experiments, we used a five-layer DNN in which the number of neurons in each layer was 1024, 120, 60, 25, 4. Therefore, the computational complexity is almost $1\times 10^{5}$. The CNN-based sensor nodes’ authentication method needs to perform:

(12) $$B^{l}=Z^{l-1}⊗W^{l}+\xi^{l}.$$

The computational complexity of the mathematical operation of the CNN-based method is almost $O\left(\max\left(n^{1}\times n_{ker}^{1}\times n_{num}^{1},n^{2}\times n_{ker}^{2}\times n_{num}^{2},\ldots ,n_{full}^{L-1}\times n^{L}\right)\right)$, where $n^{l}$ indicates the number of convolution operations in the $l^{\mathrm{th}}$ layer. $n_{ker}^{l}$ and $n_{num}^{l}$ denote the dimensions and the number of convolution kernels in the $l^{\mathrm{th}}$ layer. $n_{full}^{L-1}$ and $n^{L}$ represent the number of neurons in the fully-connected and output layers. In our numerical experiments, we used eight convolution kernels with dimensions of 4×4×1 and 16 convolution kernels with dimensions of 2×2×8. The dimensions of the input layer and fully-connected layer were 32×32×1 and 1×1×256, respectively. Therefore, the computational complexity of the CNN-based method was almost $5\times 10^{5}$. The CPNN-based sensor nodes’ authentication method needs to perform convolution pre-processing on CSI, and the computation complexity of pre-processing was relatively small. The computational complexity of the CPNN-based method is $O\left(\max\left(n^{0}\times n_{ker}^{0}\times n_{num}^{0},n^{1}\times n^{2},\ldots ,n^{L-1}\times n^{L}\right)\right)$, which is almost the same as that of the DNN-based method, where $n^{0}$ denotes the number of convolution operations in pre-processing and $n^{l}$ denotes the dimensions of the CSI after being processed in the $l^{\mathrm{th}}$ layer. $n_{ker}^{0}$ and $n_{num}^{0}$ denote the dimension and number of convolution kernels in pre-processing, respectively. There were 16 convolution kernels of size 4×4×1 in the pre-processing of the CPNN-based method. There were four convolution steps. The computational complexity of the CPNN-based method was almost $2\times 10^{4}$.

During the retraining phase, the number of parameters that needed to be trained is shown in Table 2. The DNN-based sensor nodes’ authentication method needs to train weight matrix $W^{†}$ and threshold vector $\xi^{†}$, in which it needs to train $\left(n^{1}\times n^{2}+n^{2}\times n^{3}+\ldots +n^{L-1}\times n^{L}\right)$ parameters. There were almost $1\times 10^{5}$ parameters for the DNN-based sensor nodes’ authentication method in our numerical experiments. The CNN-based sensor nodes’ authentication method needs to train convolution kernels $W^{◊}$ and threshold vector $\xi^{◊}$, which needed to train $\left(n_{kernel}^{1}\times n_{num}^{1}+n_{kernel}^{2}\times n_{num}^{2}+n_{full}^{L-1}\times n^{L}\right)$ parameters. In our numerical experiments, only $1\times 10^{3}$ parameters needed to be trained. The CPNN-based authentication method needed to train weight matrix $W^{⊥}$ and threshold vector $\xi^{⊥}$. Like the DNN-based method, the parameters of CPNN-based method depended on the number of neurons in each layer. However, the dimension of the input in the CPNN-based method was much smaller than the DNN-based method. The number of neurons in each layer of CPNN was 256, 50, 25, 12, and 4. There were almost $1\times 10^{4}$ parameters that needed to be trained in the retraining phase.

## 5. Numerical Experiments

Simulations have been performed to evaluate the performance of DL-based PHY-layer authentication for industrial wireless sensor networks. We performed the simulations under different nodes and analyzed the impact of the number of sensor nodes on the authentication success rate. We also compared the performance of different algorithms under different numbers of sensor nodes. Cost *J* denotes the value of the loss function, which is calculated by (6) or (7). The authentication rate $P_{a}$ is defined as the probability of discriminating the wireless sensor nodes.

We considered the tapped delay line (TDL) model to simulate Raleigh fading channels with multipath delays [32]. The TDL model uses a set of non-frequency selective fading generators (such as the FWGN model or the Jakes model), where each generator is independent of each other and has an average power of one. The channel state information of different transmitters can be generated by:

(13) $$y\left(n\right)=\sum_{d=0}^{N_{D}-1}h_{d}\left(n\right)x\left(n-d\right).$$

where $N_{D}$ denotes the number of taps of the filters. We set the normalized Doppler shift $f_{d}=0.125$, and six paths with different power delays were selected to synthesize the channels of different wireless sensor nodes. For more realistic consideration, the time delay of the first five paths of the sensor nodes was the same, which was 0 second (s), $5\times 10^{-6}$ s, $1\times 10^{-5}$ s, $1.5\times 10^{-5}$ s, and $2\times 10^{-5}$ s, respectively. When there were twelve sensor nodes, the time delay of the sixth path of each sensor node was as shown in Table 3.

When there were four sensor nodes, the sixth paths of each sensor node were $6.6\times 10^{-5}$ s, $4.6\times 10^{-5}$ s, $3.4\times 10^{-5}$ s, $2.2\times 10^{-5}$ s, respectively. Sampling interval $t_{sampling}=5\times 10^{-6}$ s; the signal to noise ratio (SNR) of the simulation channel was 4 dB; the number of subcarriers was $n_{sub\_carrier}=256$; the number of pilot intervals and of the cyclic prefix length were $n_{pilot\_inteval}=256$ and $l_{cp\_length}=30$, respectively.

We used a five-layer neural network for the DNN-based sensor nodes’ authentication method, where the numbers of neurons in the hidden layer were 120, 60, and 25. The size of the input layer was determined by the CSI dimension, and the size of the output layer was determined by the number of sensor nodes. The convolutional neural network used in the CNN-based algorithm had seven layers, which were an input layer, two convolution layers, two pooling layers, one fully-connected layer, and an output layer. The two convolutional layers respectively used eight convolution kernels of size 4×4×1 and 16 convolution kernels of size 2×2×8, respectively. For the CPNN-based algorithm, it had 16 convolution kernels of size 4×4×1 for the convolution pre-processing. In the authentication phase and retraining phase, we used a five-layer neural network for the CPNN-based algorithm, where the numbers of neurons in the hidden layer were 50, 25, and 12. Moreover, the adaptive moment estimation (Adam) accelerated gradient algorithm and minibatch skill was used for the accelerating of the neural networks’ training.

**Figure 5:**
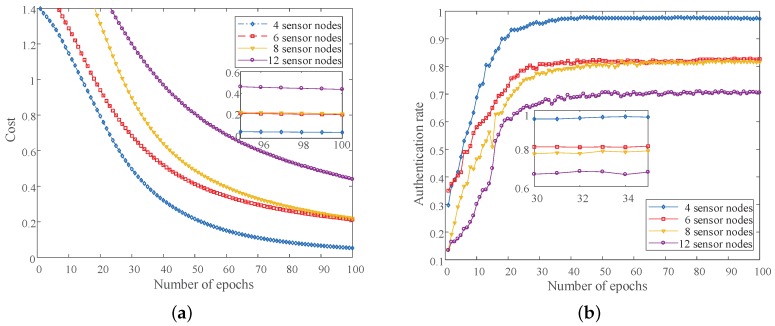
The authentication performance with different sensor nodes. (**a**) The cost value under different numbers of sensor nodes with the DNN-based method; (**b**) The authentication rate under different numbers of sensor nodes with the DNN-based method.

As shown in Figure 5a, the x-axis is the number of neural network iterations and the y-axis is the cost function value. As the number of iterations increased, the cost function value decreased. In addition, the fewer the sensor nodes, the faster the cost function value dropped. We can visually see the authentication rate under different sensor nodes from Figure 5b. After 30 iterations, the authenticate rates tended to be stable. However, the authentication rate of four sensor nodes was higher than that of six sensor nodes, and the authentication rate of 12 sensor nodes was the lowest. Specifically, after 30 iterations, the authentication rates of 4 sensor nodes, 6 sensor nodes, 8 sensor nodes, and 12 sensor nodes was 95.5%, 80.83%, 77.25%, and 66.5%, respectively.

**Figure 6:**
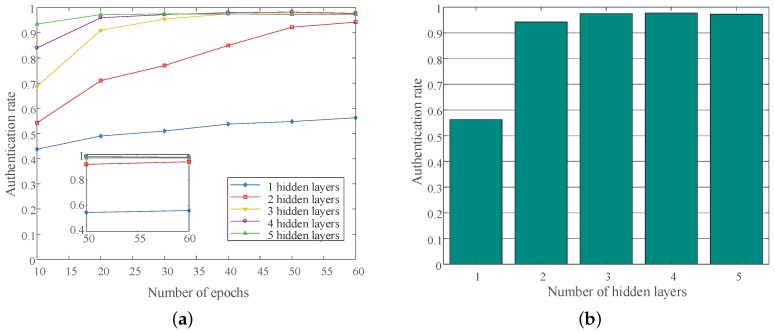
The authentication performance with different numbers of hidden layers. (**a**) The authentication rate of different numbers of hidden layers; (**b**) The authentication rate of different numbers of hidden layers after training was stabilized.

By discussing the authentication success rate under different numbers of hidden layers, we researched the robustness of the DL-based authentication method. The DNN-based algorithm had the most excellent performance. Therefore, we considered the influence of different hidden layer numbers on the authentication rate under the DNN-based method. As shown in Figure 6a, the authentication rate of the DNN-based method with different numbers of hidden layers increased as the iterations increased. The greater the number of hidden layers, the faster the convergence of the neural network’s performance. The authentication success rate of the DNN-based method with different hidden layers after the training was stabilized are shown in Figure 6b. As the number of hidden layers increased, the authentication success rate increased. However, due to the inherent characteristics of the specific wireless channels, the performance of the DNN-based method did not continue to grow and tended to be stable, after the number of hidden layers was increased to a certain number.

**Figure 7:**
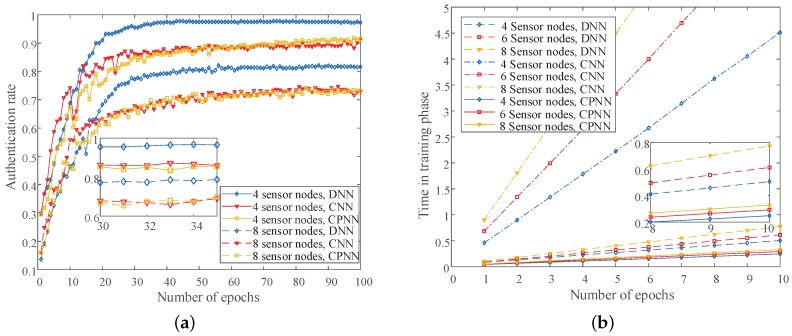
The authentication performance with different algorithms. (**a**) The authentication rate of different algorithms under different numbers of sensor nodes; (**b**) The time in the training phase of different algorithms under different numbers of sensor nodes.

In addition, we performed simulation analysis on the authentication performance of different algorithms under different numbers of sensor nodes. As shown in Figure 7a, the DNN-based method had the best performance, because it had many parameters. For example, the authentication rates of the DNN-based method were 95.5% and 77.25% under four sensor nodes and eight sensor nodes, respectively. The CNN-based algorithm had the worst performance, because of the convolution and pooling and more or less lost some information of CSIs. For example, the authentication rates of the CNN-based method were 86.25% and 67.87% under four sensor nodes and eight sensor nodes. Another CPNN-based method we proposed in this paper was similar in performance to the CNN-based method. The authentication rates of CPNN-based algorithm were 85.25% and 66.75% under four sensor nodes and eight sensor nodes. However, the CPNN-based method had the shortest training time compared to the DNN-based algorithm and CNN-based algorithm, as shown in Figure 7b. Therefore, it has a better application prospect in the actual industrial wireless sensor network. We can see that the CNN-based method had the longest training time, followed by the DNN-based method.

In summary, the DNN-based sensor nodes’ authentication had the best authenticate performance and a relatively limited training time. However, its training parameters will grow exponentially as the dimensions of CSI become larger. Therefore, the DNN-based algorithm is suitable for a shorter CSI authentication scheme. The CNN-based sensor nodes’ authentication method effectively reduced the parameters that the neural network needed to train. However, due to the convolution operation and the pooling operation, it did not meet the requirements of saving training time, especially when the dimension of CSI was relatively small. At last, the CPNN-based sensor nodes’ authentication method can effectively solve the problem of training time and authentication performance. It has an unparalleled advantage in practical industrial wireless sensor network applications.

## 6. Experiments In Practical Environment

**Figure 8:**
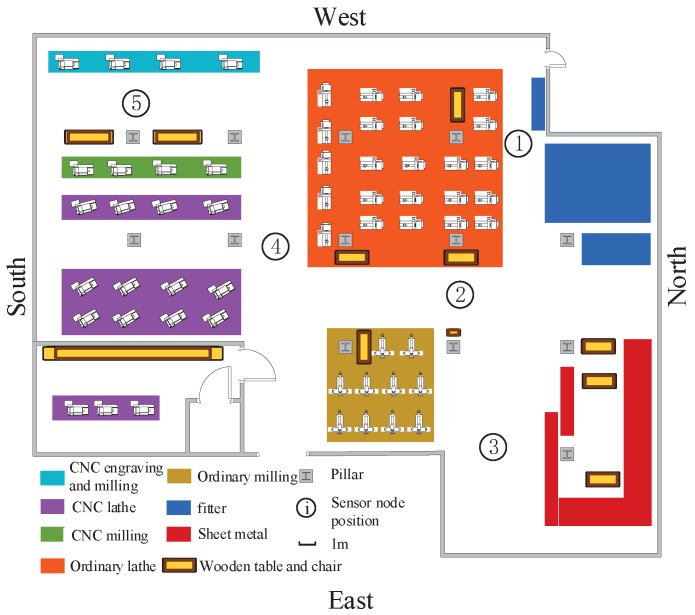
The network topology.

**Figure 9:**
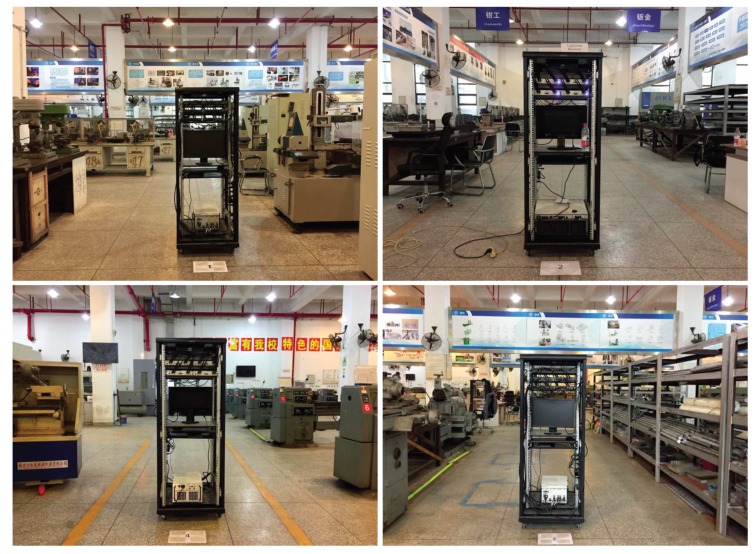
The location of the wireless sensor nodes in the practical industrial scenario.

Experiments have been performed with universal software radio peripherals (USRPs) to evaluate the authentication performance of the proposed DL-based PHY-layer authentication algorithms in industrial wireless sensor networks. The experimental simulations were performed at the school’s engineering center, which has a large number of industrial facilities, such as computer numerical control (CNC) engraving and milling, CNC lathe, and so on. As shown in Figure 8, five radio sensor nodes equipped with industrial computer and USRPs were placed in a $43.56\times 38.84\times 6.5m^{3}$ factory. The base station was equipped with 8 antennas in Position 2, and the other sensor nodes were equipped with 2, 4, or 8 antennas in Positions 1, 3, 4, and 5. The distances between sensor nodes and the base station varied from 5–25 meters (m). In this experiment, we set the carrier frequency $f_{c}=3$ gigahertz (GHz), the interval of subcarriers $f_{interval\_subcarrier}=15$ kilohertz (kHz), and the number of subcarriers $n_{subcarrier}=128$. The transmitting power of USRPs was 15 dBm, and the transmission gain was 20 dB. The practical view of the engineering center is shown in Figure 9.

**Figure 10:**
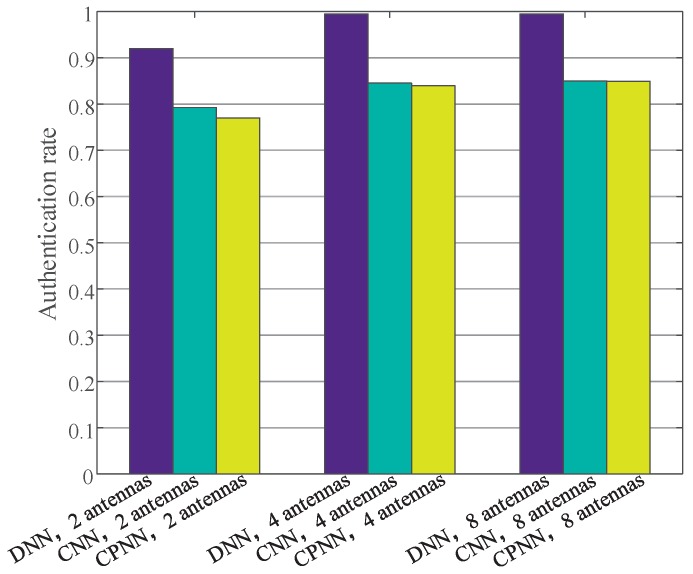
The authentication rate with USRPs.

We tested the authentication rates of sensor nodes with different antennas in different locations. As shown in Figure 10, as the number of antennas increased, the authentication success rate increased correspondingly. For example, the authentication rate of the DNN-based algorithm with 2 antennas was 92%, while the authentication rate of the DNN-based algorithm with 4 antennas and 8 antennas was 99.5% and 99.5%, respectively. From the histograms of different colors, we can see that the DNN-based sensor nodes’ authentication method had the best performance. For example, the authentication rate of DNN-based algorithm with 8 antennas was 99.5%, while the authentication rate of the CNN-based algorithm with 8 antennas was 85%. In addition, the CPNN-based algorithm had the same performance as the CNN-based algorithm. However, the retraining time of the CPNN-based method was much shorter than that of the CNN-based algorithm.

## 7. Conclusions

The DL-based PHY-layer authentication method in industrial wireless sensor networks we proposed in this paper has a strong practical significance. It can both achieve lightweight authentication and authenticate multiple nodes simultaneously. Especially for the CPNN-based sensor nodes’ authentication algorithm, it had good authentication performance and an ultra-short retraining time. The DNN-based sensor nodes’ authentication had the best authenticate performance and a relatively limited training time. However, its training parameters will grow exponentially as the dimensions of CSI become larger. Therefore, the DNN-based algorithm is suitable for a shorter CSI authentication scheme. As shown in Table 2, the CNN-based algorithm and CPNN-based algorithm effectively reduced the parameters that the neural network needed to train. However, due to the convolution operation and the pooling operation, the CNN-based algorithm did not meet the requirements of saving training time, especially when the dimension of CSI was relatively small. At last, the CPNN-based sensor nodes’ authentication method can effectively solve the problem of training time and authentication performance. It has an unparalleled advantage in practical industrial wireless sensor network applications.

## Figures and Tables

**Table 1:** The computational complexity in the authentication phase.

Algorithms	Computational Complexity	Simulation
DNN-based	$O\left(\max\left(n^{1}\times n^{2},n^{2}\times n^{3},\ldots ,n^{L-1}\times n^{L}\right)\right)$	$1\times 10^{5}$
CNN-based	$O\left(\max\left(n^{1}\times n_{ker}^{1}\times n_{num}^{1},n^{2}\times n_{ker}^{2}\times n_{num}^{2},\ldots ,n_{full}^{L-1}\times n^{L}\right)\right)$	$5\times 10^{5}$
CPNN-based	$O\left(\max\left(n^{0}\times n_{ker}^{0}\times n_{num}^{0},n^{1}\times n^{2},\ldots ,n^{L-1}\times n^{L}\right)\right)$	$2\times 10^{4}$

**Table 2:** The number of parameters in the retraining phase.

Algorithms	Number of Parameters	Simulation
DNN-based	$\left(n^{1}\times n^{2}+n^{2}\times n^{3}+\ldots +n^{L-1}\times n^{L}\right)$	$1\times 10^{5}$
CNN-based	$\left(n_{kernel}^{1}\times n_{num}^{1}+n_{kernel}^{2}\times n_{num}^{2}+n_{full}^{L-1}\times n^{L}\right)$	$1\times 10^{3}$
CPNN-based	$\left(n^{1}\times n^{2}+n^{2}\times n^{3}+\ldots +n^{L-1}\times n^{L}\right)$	$1\times 10^{4}$

**Table 3:** The time delay of the sixth path of 12 sensor nodes.

**Sensor Node 1**	**Sensor Node 2**	**Sensor Node 3**	**Sensor Node 4**	**Sensor Node 5**	**Sensor Node 6**
$6.6\times 10^{-5}$ s	$6.2\times 10^{-5}$ s	$5.8\times 10^{-5}$ s	$5.4\times 10^{-5}$ s	$5.0\times 10^{-5}$ s	$4.6\times 10^{-5}$ s
**Sensor Node 7**	**Sensor Node 8**	**Sensor Node 9**	**Sensor Node 10**	**Sensor Node 11**	**Sensor Node 12**
$4.2\times 10^{-5}$ s	$3.8\times 10^{-5}$ s	$3.4\times 10^{-5}$ s	$3.0\times 10^{-5}$ s	$2.6\times 10^{-5}$ s	$2.2\times 10^{-5}$ s

## Abbreviations

The following abbreviations are used in this manuscript:

AKA	Authentication and key agreement
ANN	Artificial neural network
CNN	Convolutional neural network
CPNN	Convolution preprocessing neural network
CSI	Channel state information
DL	Deep learning
DNN	Deep neural network
EAP	Extensible authentication protocol
IWSNs	Industrial wireless sensor networks
MHz	Megahertz
OFDM	Orthogonal frequency division multiplexing
PHY	Physical
QoS	Quality of service
ReLU	Rectified linear unit
RSS	Received signal strength
RSSI	Received signal strength indicator
TDD	Time division duplexing
USRPs	Universal software radio peripherals
WSNs	Wireless sensor networks
